# Factors related to the appearance and development of burnout in nursing students: a systematic review and meta-analysis

**DOI:** 10.3389/fpubh.2023.1142576

**Published:** 2023-05-04

**Authors:** Almudena Velando-Soriano, Nora Suleiman-Martos, Laura Pradas-Hernández, María José Membrive-Jiménez, Lucia Ramírez-Baena, Jose L. Gómez-Urquiza, Guillermo Arturo Cañadas-De La Fuente

**Affiliations:** ^1^Hospital Universitario San Cecilio, Servicio Andaluz de Salud, Granada, Spain; ^2^Faculty of Health Sciences, University of Granada, Granada, Spain; ^3^Faculty of Health Sciences of Ceuta, University of Granada, Cueta, Spain

**Keywords:** burnout, prevalence, meta-analysis, nursing student, COVID-19

## Abstract

**Introduction:**

Burnout may be suffered not only by experienced nurses, but also by those in training. The university environment can be highly stressful for student nurses, who are exposed to various stress-producing situations.

**Aim:**

The aim of this study is to identify and analyse the main risk factors for burnout among nursing students.

**Methods:**

A systematic review with meta-analysis was performed. The search equation used was “Burnout AND Nursing students”. Quantitative primary studies on burnout in nursing students, and related risk factors published in English or Spanish and with no restriction by year of publication were included.

**Results:**

A sample of n = 33 studies were included. Three variables are identified can influence burnout in nursing students: academic, interpersonal, environmental and/or social factors. The meta-analyses, with the higher sample of n = 418 nursing students, show that some personality factors, empathy, and resilience are correlated with emotional exhaustion, depersonalization and personal accomplishment.

**Conclusion:**

The personality factors that can influence the development of burnout in nursing students, such as resilience and empathy, among others, must be taken into account when preventing the appearance and treating burnout. Professors should teach nursing students to prevent and recognize the most frequent symptoms of burnout syndrome.

## 1. Introduction

Burnout Syndrome is increasingly present in society. It affects various population groups but has a particular impact on health professionals (1). The syndrome is caused by long-term, continual exposure to work stressors, which produce negative effects both on the persons affected and on their environment (2).

According to Maslach and Jackson (3), burnout syndrome consists of three dimensions: Emotional Exhaustion (EE), Depersonalisation (D) and (low levels of) Personal Accomplishment (PA). The persons affected are mainly those whose work involves dealing with other people, as is the case with health workers. The three dimensions of the syndrome are usually measured using validated scales such as the Maslach Burnout Inventory (3).

This syndrome is considered an occupational disease, since it can provoke various health problems, such as anxiety and depression. Among the health professionals affected, nurses are especially vulnerable, as they are exposed to a wide range of sociodemographic, psychological and work-related risk factors (4–8). Relevant sociodemographic variables include gender, age, marital status and number of children (9). Among the psychological variables to be considered, personality traits and characteristics are of great importance, causing different people to react in diverse ways to stressful situations (8). Work-related variables are perhaps the most studied in this context. These variables concern all aspects of the individual's employment, including the workplace setting, salaries, relationships with co-workers and job satisfaction (10).

Nurses work in an environment involving complex relationships and regular contact with the pain and death of patients and with the suffering of their families. Moreover, they may be affected by conflicts, both at the organizational level with co-workers and managers, and also with patients and their families. All these factors heighten nurses' vulnerability to burnout syndrome, a problem that directly affects the care provided, reducing productivity and diverting attention from patients (11). Studies have shown that the specific area of health care in which nurses are employed is an important factor in determining the likelihood of burnout developing; thus, burnout is especially prevalent among personnel working in the emergency department (12), in primary care (13) or in the medical area (14).

An interesting question to address in this respect is whether trainee nurses are also affected by this problem. If so, it would impact on their behavior, hinder learning and reduce academic performance. Problems such as a gap between expectations and clinical reality or a perceived lack of support from mentors can lead to academic failure and dropout (15).

Since the COVID-19 pandemic began, the greatly increased use of online platforms has heightened the risk of nursing students developing burnout, by limiting their contact with clinical cases (16). Ultimately, the presence of burnout would prejudice the student's subsequent career, restricting the skills and qualifications obtained and leading to disillusionment with the profession (17, 18). The proper management of factors related to burnout would help prevent its appearance and enable trainee nurses to better adapt to the clinical environment (19, 20).

In view of these considerations, the aim of this study is to identify and analyse the main risk factors for burnout among nursing students.

## 2. Materials and methods

A systematic review with meta-analysis was performed.

### 2.1. Search strategy

A bibliographic search was carried out in the PubMed, Scopus and CINAHL databases in April 2021. The search equation used was (“burnout” OR “burnout syndrome”) AND (“undergraduate nurses” OR “nursing students”). Equation descriptors were obtained from the Medical Subject Headings (MeSH) thesaurus. For the collection and analysis of data, the PRISMA recommendations for systematic reviews (21) were followed.

### 2.2. Inclusion and exclusion criteria

The following inclusion criteria were applied: (a) quantitative primary articles on burnout in nursing students, and related risk factors; (b) published in English or Spanish; (c) no restriction by year of publication.

Studies based on mixed samples, in which independent results for nursing students were not provided, were excluded from consideration.

### 2.3. Selection of articles and information analysis

The literature search process was carried out in three phases. In the first, three of the authors selected appropriate papers according to the title and abstract in each case, ensuring that duplicate articles and those not related to the subject of study were eliminated. In the second phase, the same authors performed a critical reading of the articles. Finally, each of the remaining publications was examined to detect possible methodological bias. At all times, these authors worked independently. Any disagreements were resolved by consensus and consultation with a fourth author. 24 meta-analysis were performed to calculate the effect size (correlation) between burnout dimensions and other variables. The software used for the analysis was StatsDirect (StatsDirect Ltd, Cambridge, UK). The heterogeneity analysis was performed using I2 value and publication bias was assessed using the Egger linear regression test.

### 2.4. Study data

The following study variables were considered: (a) bibliographic data (author, year, country); (b) study design; (c) sample; (d) measurement instruments used; (e) mean and standard deviation of level of burnout, in each of its dimensions; (f) significant relationships among the factors studied.

### 2.5. Assessing study quality and determining bias

The quality of each article was evaluated by three independent authors, who consulted a fourth author if any disagreement arose. The level of evidence was analysed according to the recommendations of the Oxford Center for Evidence-Based Medicine (22). These findings are summarized in the Tables 1, 2.

**Table 1:** Main results included in the review (studies using MBI as a measurement instrument).

**References and Country**	**Design**	**Sample (% response rate)**	**Measurement instrument**	**Results. Mean (SD) OF EE, D And PA. burnout and related variables**		**EL**	**RG**
McKee-Lopez et al. (USA) (23)	Cross-sectional study	*N =* 211	MBI; PHQ9	ACE scores and EE (*p <* 0.001); Depression severity and EE (*p <* 0.01); D (*p <* 0.01)		2c	B
Naderi et al. (Iran) (24)	Cross–sectional study	*N =* 337 (83.83%)	MBI–SS; NFCS; SGSES	EE 2.62 (1.20); D 2.40 (1.29); PA 2.53 (0.97); CORRELATIONS: EE and Need for cognition: −0.192 *P* ≤ 0.001; Self efficacy: −0.224 *P* ≤ 0.001	D and Need for cognition: −0.208 *P* ≤ 0.001; Self efficacy: −0.300 *P* ≤ 0.001; PA and Need for cognition: −0.318 *P* ≤ 0.001; Self efficacy: −0.458 *P* ≤ 0.001	2c	B
Quina–Galdino et al. (Brazil) (25)	Cross–sectional study	*N =* 114 (95.79%)	MBI–SS	Multiple correlation; EE and Academic year (p = 0.003); Dissatisfaction with the course (p = 0.049); D and Academic year (*p <* 0.001); and Homework workload (*p <* 0.001); Dissatisfaction with the course (p = 0.001); PA and Academic year (p = 0.012); Homework workload (p = 0.042); Burnout and Homework workload (p = 0.001)		2c	B
Ríos–Risquez et al. (Spain) (26)	Cross–sectional study	*N =* 218	MBI–GS; CD–RISC	EE 2.43 (1.09); D 1.65 (1.17); PA 4.23 (0.69); Pearson correlation: EE and Resilience (*r =* −0.248) *p* ≤ 0.01; Relationship with teachers *p* ≤ 0.01; PA and Relationship with teachers *p* ≤ 0.01; Resilience (*r =* 0.521) *p* ≤ 0.01		2c	B
Ríos–Risquez et al. (Spain) (27)	Cross–sectional study	*N =* 113(97.41%)	MBI–SS; CD–RISC; GHQ−12	Pearson correlation: EE and Resilience *r =* −0.51 *p* ≤ 0.001; Age *r =* 0.25 *p* ≤ 0.01; Psychological discomfort *r =* −0.62 *p* ≤ 0.001. D and Resilience *r =* −0.20 *p* ≤ 0.05. PA and Resilience *r =* 0.35 *p* ≤ 0.001; Psychological discomfort *r =* 0.30 *p* ≤ 0.01		2c	B
Rohmani and Andiani (Indonesia) (28)	Cross–sectional study	*N =* 69	MBI–SS	Academic self–efficacy and burnout (*r =* −0.884) *p* ≤ 0.001; Gamma correlation; EE and Academic self–efficacy (*r =* −0.898) *p* ≤ 0.001	D and Academic self–efficacy (*r =* −0.873) *p* ≤ 0.001; PA and Academic self–efficacy (*r =* −0.792) *p* ≤ 0.001; Most students with low self–efficacy experience severe burnout.	2c	B
Tomaschewski–Barlem et al. (Brazil) (29)	Cross–sectional study	*N =* 168	MBI–SS	PA and Age *r =* 4.64 *p* = 0.042; and Work *r =* 4.64 *p* = 0.042; and Performs leisure activity and work *r =* 4.63 *p* = 0.009; Academic year *r =* 4.37 *p* = 0.05; Extracurricular activity *r =* 4.63 *p* = 0.041; Intention to withdraw from the course *r =* 4.26 *p* = 0.016. EE and Work *r =* 4.64 *p* = 0.042; Intention to withdraw from the course *r =* 2.22 *p* = 0.005. D and Work *r =* 4.64 *p* = 0.042		2c	B
Valero– Chillerón et al. (Spain) (30)	Cross–sectional study	*N =* 126	MBI–SS; KEZKAK questionnaire	EE and Academic Year *p* = 0.007.; EE and Satisfaction with clinical clerkship: < *p* = 0.001	D and Academic year *p* = 0.027; PA and Satisfaction with clinical clerkship *p* = 0.003	2c	B
De Vasconcelos et al. (Brazil) (31)	Cross–sectional study	*N =* 100	MBI–SS	Univariate Logistic Regression; Academic year and Burnout *p* = 0.036; Use of Medication and Burnout *p* = 0.002; Thinking of dropping out of the course and Burnout *p* = 0.001	Multivariate Logistic Regression; Thinking of dropping out of the course and Burnout *p* = 0.025	2c	B
Watson et al. (United Kingdom) (32)	Cohort study	*N =* 147 (93%)	MBI; NEO–FFI; GHQ−12; CISS−21; SINE	Wave 1: EE 22.6 (7.7); D 10.4 (4.3); PA 29.1 (6.2)	Wave 2: EE 24.5 (9.2); D 11.5 (4.1); PA 27.0 (6.1)	2b	B
				Correlations; EE and Neuroticism (*r =* 0.52) *p* ≥ 0.01, Extraversion (*r =* −0.32) *p* ≥ 0.01; Conscientiousness (*r =* −0.21) *p* ≥ 0.01; Coping emotion (*r =* 0.42) *p* ≥ 0.01; PA and Extraversion (*r =* 0.28) *p* ≥ 0.01; Openness (*r =* −0.19) *p* ≥ 0.05; Conscientiousness (*r =* 0.18) *p* ≥ 0.05; Coping with task (*r =* 0.40) *p* ≥ 0.01; D and Neuroticism (*r =* 0.28) *p* ≥ 0.01; Extraversion (*r =* −0.19) *p* ≥ 0.01; Openness (*r =* −0.23) *p* ≥ 0.01,	Agreeableness (*r =* −0.17) *p* ≥ 0.01 and Conscientiousness (*r =* 0.21) *p* ≥ 0.01; D and Coping emotion (*r =* 0.41) *p* ≥ 0.01; Multiple Regression; EE and Neuroticism β = 0.443 *p* ≥ 0.001; EE and Emotion Coping β = 0.224 *p* = 0.049; D and Emotion Coping β = 0.269 *p* = 0.014; D and Agreeableness β = −0.230 *p* = 0.035; PA and Neuroticism β = −0.273 *p* = 0.012		
Babenko et al. (Canada) (33)	Cross–sectional study	*N =* 126	CIS; Subscales of MBI–GS: CE and D	EE 2.91 (1.38); D 1.25 (1.15) Correlations: EE and Nurse Incivility: *r = 0.42 p ≤ 0.001;* Clinical instructor incivility: *r = 0.24 p ≤ 0.05;* Healthcare professionals' incivility: *r = 0.18 p ≤ 0.05*		2c	B
Batista et al. (Brazil) (34)	Cross–sectional study	*N =* 301	MBI–HSS; NSSS	Correlations: Significant association between burnout and low academic satisfaction: Burnout and Curriculum and teaching: *p = 0.009;* Professional social interaction: p * ≤ 0.001* (the less interaction, the higher the burnout); Teaching environment: p *= 0.008* (higher burnout if worse environment).		2c	B
Bolaños and Rodríguez (Costa Rica) (35)	Cross–sectional study	*N =* 289	MBI–SS	Men suffer less burnout than women (22.7% vs 8.7%)		2c	B
Chust–Hernández et al. (Spain) (36)	Cross–sectional study	*N =* 494	MBI–SS; CAEX; SWLS	Mean burnout: 28.4 (11.16); Correlations: Burnout and Self–steem: *r = −0.28 p = 0.01;* Anxiety: *r = 0.44 p = 0.01;* Anxiety about exams: *r = 0.4 p = 0.01;* Satisfaction with life: *r = 0.33 p = 0.01;* Sleep satisfaction *r = 0.4 p = 0.01*		2c	B
Da Silva (Brazil) (37)	Cross–sectional study	*N =* 570; (78%)	MBI–SS; Hardiness Scale	EE 3.57 (1.31); D 1.78 (1.29); PA 2.12 (0.82); Correlations: Burnout and Hardy personality: *p = 0.033*. If hardy personality less burnout		2c	B
Deary et al. (United Kingdom) (38)	Longitudinal cohort study	N ST1 = 139 (100%); N ST2 =; 111 (80%); N ST3 =; 76 (55%)	MBI; GHQ−28; NEO–FFI; AH−4	ST2: EE: 15 (7.2); D: 3.9 (4.1); PA: 37.1 (6.5); Correlations; D and Agreeableness: −0.19 *p < * 0.05; PA and Conscientiousness: 0.18 *p < 0.05*	EE and Neuroticism: 0.31 *P < 0.01;* CISS emotion–oriented coping: 0.21 *p < * 0.05; Stress: 0.36 *p <* 0.01; Confidence: 0.30 *p <* 0.01; Financial Stress: 0.23 *p <* 0.05; Educational Stress: 0.39 *p <* 0.01	2+	B
				ST3: EE: 13.99 (7.7); D: 3.5 (3.9); PA: 39 (5.1); Correlations; EE and Clinical stress: 0.23 *p < 0.05*	D and Openness: 0.29 *p <* 0.01; Conscientiousness: −0.37 *p <* 0.05; PA and Conscientiousness: 0.20 *p <* 0.05		
Frögéli et al. (Sweden) (39)	Pre–post study or clinical trial	*N =* 113; N post intervention = 80; N 3 months follow up = 63	MBI; AFQ–Y; MAAS; PSS	Control Group: Pre: 2.3 (0.2); Post: 2.4 (0.1); 3 months later: 2.1 (0.2)	Intervention Group: Pre: 2.5 (0.1); Post: 2.4 (0.1);; 3 months later: 2.5 (0.1)	1b	A
				After the intervention of acceptance and commitment training techniques, the intervention group showed lower levels of burnout, although without statistically significant data p = 0.33.			
Gibbons et al. (United Kingdom) (40)	Cross–sectional study	*N =* 171 (61%)	MBI; GSE; The Brief COPE; SNSI	Correlations: EE and Learning and teaching hassles (*r =* 0.329) *, learning and teaching uplifts (*r =* −0.301) *, placement related hassles (*r =* 0.252) *, placement–related uplifts (*r =* −0.215) *, course organization hassles (*r =* 0.275)*, support hassles (*r =* 0.231)*, support uplifts (*r =* −0.299)*, dispositional control (*r =* −0.505)*, context control (*r =* −0.456)*, avoidance coping (*r =* 0.521)*, seeking support (0.199)*.; D and Learning and teaching hassles (*r =* 0.239) *, placement related hassles (*r =* 0.305) *, course organization hassles (*r =* 0.255) *, support hassles (*r =* 0.307) *, approach coping (*r =* 0.322) *, avoidance coping (*r =* 0.296) *, altering consciousness (*r =* 0.286) *, seeking support (0.208) *.; PA and Learning and teaching hassles (*r =* −0.282) *, learning and teaching uplifts (*r =* 0.229) *, placement related hassles (*r =* −0.179) *, placement–related uplifts (*r =* 0.222) *, course organization hassles (*r =* −0.172) *		2c	B
Haack MR.; (USA) (41)	Cross–sectional study	*N =* 367; Wave I = 272; Wave II = 307; Wave III = 128	MBI; MOS–SSS	EE 23.3 (9.6); D 5.5 (4.7); RP 32.1 (6.5); Correlations: Increased EE and D from the 2nd year to the last in nursing school *p* ≤ 0.05. EE and Faculty Interaction *r =* −0.21 *p* ≤ 0.01	D and External/Others *r =* 0.23 *p* ≤ 0.01; External Setting *r =* 0.35 *p* ≤ 0.01; PA and Emotional Support: *r =* 0.29 *p* ≤ 0.01PA and Internal/self *r =* 0.22 *p* ≤ 0.01	2c	B
Katsifaraki and Tucker. (United Kingdom) (42)	Cross–sectional study	*N =* 183	MBI–HSS; TAS−20; AWS; BDI–II; COPE Dispositional Inventory	EE = 14.22 (9.51); D = 4.12 (4.05); PA = 34.56 (9.55); Correlations: EE and Workload *r =* −0.58 *p* ≤ 0.001; Control over the workplace *r =* −0.21 *p* ≤ 0.01; Rewards *r =* −0.22 *p* ≤ 0.01; Sense of Community *r =* −0.28 *p* ≤ 0.001; Discrepancy of values *r =* −0.17 *p* ≤ 0.05; Depression *r =* 0.41 *p* ≤ 0.01; D and Workload *r =* −0.23 *p* ≤ 0.01; Sense of Community *r =* −0.17 *p* ≤ 0.05;	D and Discrepancy of values *r =* −0.19 *p* ≤ 0.01; Depression *r =* 0.25 *p* ≤ 0.01; Task coping *r =* −0.13 *p* ≤ 0.05; Avoidance *r =* 0.22 *p* ≤ 0.01; Difficulty identifying feelings *r =* 0.31 *p* ≤ 0.001; PA and Workload *r =* 0.15 *p* ≤ 0.05; Values *r =* 0.13 *p* ≤ 0.05; Depression *r =* −0.23 *p* ≤ 0.01; Task coping *r =* 0.21 *p* ≤ 0.01; Social coping *r =* −0.13 *p* ≤ 0.05; Avoidance *r =* −0.16 *p* ≤ 0.05; Difficulty identifying feelings *r =* −0.16 *p* ≤ 0.05	2c	B
Liébana–Presa C et al. (Spain) (43)	Cross–sectional study	*N =* 134	SSIE; SEIS; EIE−25; MBI–SS	EE 2.8(1.2); D 1.1 (0.99); PA 4.1 (0.9); Pearson correlation: PA and Emotional intelligence = 0.419 *p* ≤ 0.01; Conscience *r =* 0.451 *p* ≤ 0.01; Control *r =* 0.203 *p* ≤ 0.01; Empathy *r =* 0.259 *p* ≤ 0.01; Motivation *r =* 0.228 *p* ≤ 0.01; Social skills *r =* 0.306 *p* ≤ 0.01; D and Motivation *r =* −0.228 *p* ≤ 0.01		2c	B
Lopes and Nihei. (Brazil) (17)	Cross–sectional study	*N =* 284 (68.3%)	MBI; IRI–Occupational; SS–SF	Spearman correlation: EE and Self–Efficacy: −0.214 *p <* 0.001; D and Empathic concern: −0.312 *p <* 0.001; Perspective taking: −0.218 *p <* 0.001; PA and Empathic concern: 0.178 *p <* 0.05; Perspective taking: −0.178 *p <* 0.05; Self efficacy: 0.428 *p <* 0.001; Personal distress: −0.270 *p <* 0.001	Multivariate Logistic Regression: High burnout and Weekly workload: 0.041 *p <* 0.001; (OR = 2.939(1.025–8.429) p = 0.040); Physical activity: 0.031 *p <* 0.001 (OR = 0.208 (0.045–0.956) p = 0.018); Personal distress: 0.068 *p <* 0.001; Empathic concern: 0.007 *p <* 0.001 (OR = 0.866(0.778–0.965) p = 0.001)	2c	B
Manzano–García et al. (Spain) (44)	Cross–sectional study	*N =* 166	MBI–SS	Regression: Burnout and Age: β = −0.21 t = −2.63 *p <* 0.001; External locus of control: β = 0.22 t = 2.81 *p <* 0.001; Perception of self efficacy: β = −0.19 t = 2.15 *p <* 0.05; Perception of economic crisis: β = 0.17 t = 2.15 *p <* 0.05		2c	B

CIS, Cortina Incivility Scale; CISS- 21, 21 Coping in Stressful Situations questionnaire; CPC, Positive Psychological Capital Scale; D, Depersonalization; EE, Emotional exhaustion; EIE-25, Escala de Inteligencia Emocional; EL, Evidence Level; GHQ−12, 12-item General Health Questionnaire; IRI- Occupational, Interpersonal Reactivity Index Occupational; MBI, Maslach Burnout Inventory; NFCS, Cacioppo and Petty's Need for Cognition Scale; NEO-FFI, NEO Five Factor Inventory; NSCI, Nurses' Self-Concept Instrument; NSSS, Nursing Student Satisfaction Scal; OLBI, Oldenburg Burnout Inventory; PA, Personal Accomplishment; PRoQOL (5), The Professional Quality of Life Scale version 5; RG, Recommendation Grade; SGSES, Sherer's General Self-Efficacy Scale; SEIS, Emotional Intelligence Scale of Schutte; SINE, Stress in Nursing Students questionnaire; SS-SF, Self-Efficacy Scale (Short Form); SWEBO, Work Engagement and Burnout.

**Table 2:** Main results included in the review (studies using others measurement instruments).

**References and Country**	**Design**	**Sample (% response rate)**	**Measurement instrument**	**Mean (SD) OF EE, D and PA**	**Results: burnout and related variables**	**EL**	**RG**
Aghajari et al. (Iran) (45)	Cross-sectional study	*N =* 223	Bersow's Academic Burnout Inventory; Newman's Learning Experience Quality Questionnaire	Mean burnout: 44.20	Correlations Academic burnout and Quality of learning experience: *r = −0.18 p = 0.006* Burnout and Learning content: *r = −0.20 p = 0.002* EE and Learning content: *r = −0.16 p = 0.015* D and Learning content: *r = −0.22 p = 0.001* EE and Learning flexibility: *r = −0.14 p = 0.03* D and Relationship with teachers: *r = −0.13 p = 0.046* Burnout and Marital status *p = 0.023* (higher burnout in single students) Burnout and Children *p = 0.02* (higher burnout in students with children) Level of interest in nursing (higher burnout in lower interest) *p ≤ 0.001*	2c	B
Akansel et al. (Turkey) (46)	Cross-sectional study	*N =* 46 (100%)	Turkish version of MBI		Correlations EE and Feeling toward the profession: *p ≤ 0.01* D and Feeling toward the profession: *p ≤ 0.05* (Significant and higher levels of EE and D in students that dislike the profession) EE and Workplace: *p ≤ 0.05*. (Higher EE if they work at inpatient clinics)	2c	B
Bulfone et al. (United Kingdom) (47)	Cross-sectional study	*N =* 1117	SWEBO; NSE-PS	-	Correlations Self-efficacy in psychomotor skills *r = −0.09 p ≤ 0.05* Higher burnout in those with moderate or low self-efficacy.	2c	B
Chamberlain et al. (Australia) (48)	Cross-sectional study	*N =* 219; (89%); N ST2 =; 111 (80%); N ST3 =; 76 (55%)	PRoQOL (5); CAMS-R; CD-RISC		Correlations Burnout and Compassion fatigue: *r = 0.5293 p < 0.001*	2c	B
				ST3; EE: 13.99 (7.7); D: 3.5 (3.9); PA: 39 (5.1)	Correlations ST3 EE and Clinical stress: 0.23 *p < 0.05* D and Openness: 0.29 *p <* 0.01; D and Conscientiousness:−0.37 *p <* 0.05 PA and Conscientiousness: 0.20 *p <* 0.05; PA and CISS task-oriented coping (*r =* 0.21) *p <* 0.05		
Kong LN et al. (China) (49)	Cross-sectional study	*N =* 1225 (86.7%)	PPS; PSES; ABS	Total: 2.80 (0.48); EE: 2.74 (0.66); D: 2.91 (0.56); Reduced PA: 2.75 (0.56)	Correlations Burnout and Sex *r =* 1.995 *p* = 0.048 Burnout and Academic Year *r =* 7.948 *p* ≤ 0.001 Burnout and Nursing as the first choice *r =* 2.590 *p* = 0.010 EE and Proactive Personality *r =*−0.264 *p* ≤ 0.01 EE and Professional Self-Efficacy *r =*−0.193 *p* ≤ 0.01 D and Proactive Personality *r =*−0.330 *p* ≤ 0.01 D and Professional Self-Efficacy *r =*−0.319 *p* ≤ 0.01 Reduced PA and Proactive Personality *r =*−0.439 *p* ≤ 0.01 Reduced PA and Professional Self-Efficacy *r =*−0.520 *p* ≤ 0.01	2c	B
Mason and Nel. (South Africa) (50)	Cross-sectional study	*N =* 153	PROQOL	18.5 (2.12)	Pearson correlation Burnout and Compassion fatigue: 0.52 *p <* 0.001 Burnout and Compassion satisfaction: −0.63 *p <* 0.001	2c	B
Njim T et al. (Cameroon) (51)	Cross-sectional study	*N =* 447	OLBI; PHQ-9	38.04 (4.78)	Significant higher score in burnout in students with depression (38.97 vs 35.91) *p* ≤ 0.01	2c	B
Nurhidayati et al. (Indonesia) (52)	Cross-sectional study	*N =* 83	BQ-B	35.50 (8.9)	No significant relations with burnout and its dimensions.	2c	B
Wang et al. (China) (53)	Cross-sectional study	*N =* 1083(95,1%)	ABS; NSCI	Burnout: 2.77 (0.53); EE: 2.72 (0.71); D: 2.97 (0.62); PA: 2.61 (0.53); Burnout and Gender *t* = 3.525 *p* = 0.000; EE and Gender *t* = 3.647 *p* = 0.000; PA and Gender *t* = 3.314 *p* = 0.001; Correlation; EE and Care *r =* −0.073 *p* ≥ 0.01; EE and Knowledge *r =* −0.334 *p* ≥ 0.01; EE and Staff relations *r =* −0.301 *p* ≥ 0.01; EE and Leadership *r =* −0.291 *p* ≥ 0.01; EE and Total professional self–concept *r =* −0.341 *p* ≥ 0.01; D and Care *r =* −0.229 *p* ≥ 0.01; D and Knowledge *r =* −0.301 *p* ≥ 0.01; D and Staff relations *r =* −0.228 *p* ≥ 0.01; D and Leadership *r =* −0.270 *p* ≥ 0.01	D and Total professional self–concept *r =* −0.297 *p* ≥ 0.01; PA and Care *r =* −0.342 *p* ≥ 0.01; Knowledge *r =* −0.403 *p* ≥ 0.01; Staff relations *r =* −0.329 *p* ≥ 0.01; Leadership *r =* −0.385 *p* ≥ 0.01; Total professional self–concept *r =* −0.420 *p* ≥ 0.01; Burnout and Care *r =* −0.328 *p* ≥ 0.01; Burnout and Knowledge *r =* −0.403 *p* ≥ 0.01; Burnout and Staff relations *r =* −0.338 *p* ≥ 0.01; Burnout and Leadership *r =* −0.364 *p* ≥ 0.01; Burnout and Total professional self–concept *r =* −0.411 *p* ≥ 0.01; Multiple Linear Regression; Burnout and Education Level B = −0.093 SD 0.031 β = −0.087 t = −3.026 *p* = 0.003	2c	B
Wang et al. (China) (54)	Cross-sectional study	*N =* 147(93%)	ABS; AES	Burnout 2.97 (0.34); EE 2.54 (0.71); D 3.51 (0.55); PA 3.02 (0.44); Academic burnout and Psychological capital *r =*−0.135 *p* ≥ 0.01; Academic engagement *r =*−0.233 *p* ≥ 0.01		2c	B

ABS, Academic Burnout Scale, AES, Academic Engagement Scale; BQ-B, Burnout Questionnaire; EE, Emotional exhaustion; EL, Evidence Level; MBI, Maslach Burnout Inventory; OLBI, Oldenburg Burnout Inventory; PA, Personal Accomplishment; PHQ9, Patient Health Questionnaire; PRoQOL (5), The Professional Quality of Life Scale version 5; RG, Recommendation Grade; SWEBO, Work Engagement and Burnout.

### 2.6. Patient and public involvement

No patient involved.

## 3. Results

**Figure 1:**
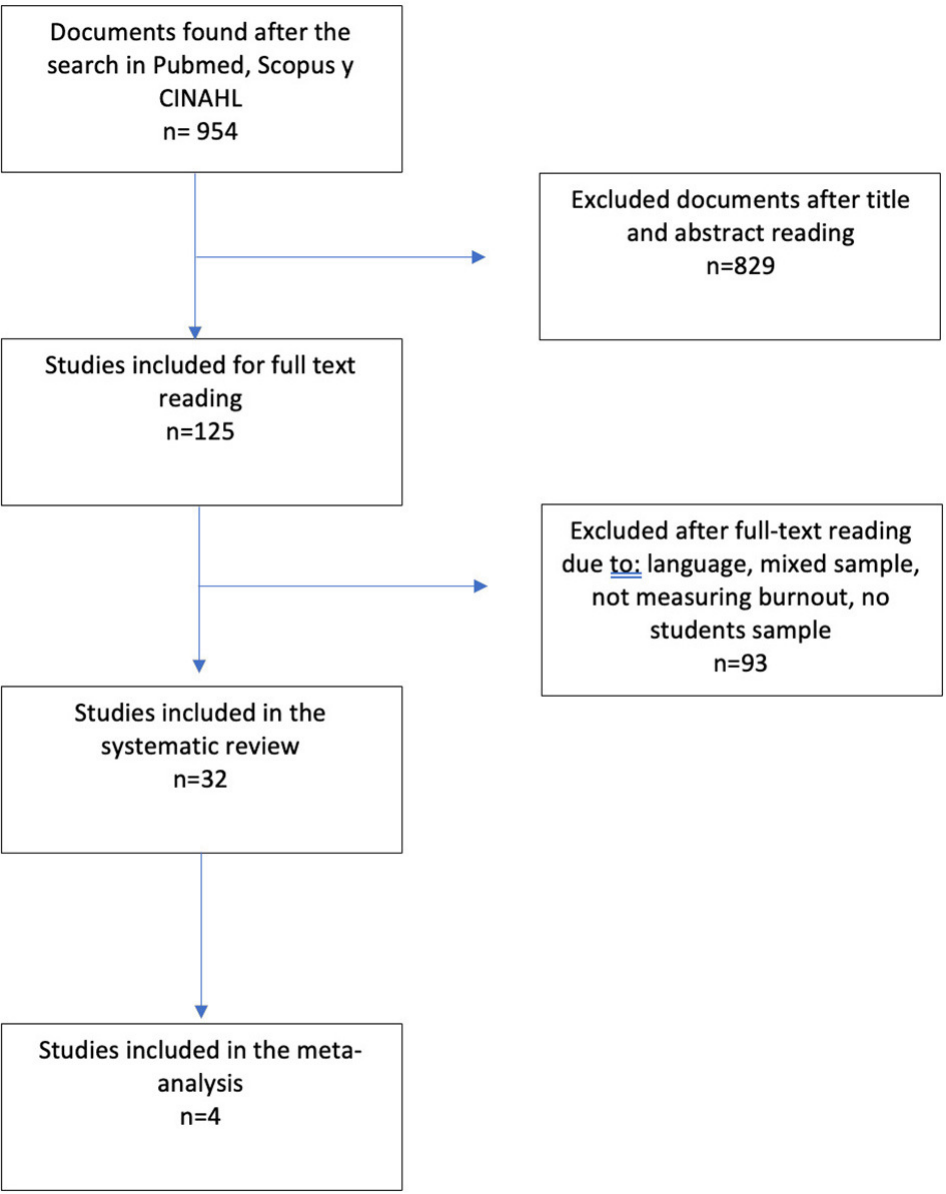
Flow chart for the selection of articles for analysis.

The literature search initially yielded 964 articles. After reading the title and abstract, and after removing duplicates, 125 remained. The full text reading then reduced this to 33 studies for the final analysis. Figure 1 shows the selection process.

### 3.1. Characteristics of the studies included in the analysis

In total, 33 articles were included in the final analysis. These studies considered a total sample population of 9,389 nursing students, mostly women. Table 1 details the main characteristics of the studies (author, year, country, sample, measurement instruments, results, degree of recommendation and level of evidence). The majority had a cross-sectional-observational design. The only exceptions were two cohort studies (32, 38) and a randomized trial (39).

With the following exceptions, the studies used a version of the Maslach Burnout Inventory to measure burnout: Aghajari et al. (45) used Bersow's Academic Burnout Inventory; Bulfone et al. (47) used the Scale of Work Engagement and Burnout (SWEBO): Chamberlain et al. (48) and Mason and Nel (50) used a version of PROQOL; Frögéli et al. (39) used the Scale of Work Engagement and Burnout; Kong et al. (49), Wang et al. (54) and Wang et al. (53) used the Academic Burnout Scale; Njim et al. (51) used the Oldenburg Burnout Inventory; and Nurhidayati et al. (52) used the Burnout Questionnaire.

### 3.2. Factors related to burnout

To simplify analysis, the burnout-related factors considered were sorted into three groups according to the classification proposed by Caballero et al. (55).

#### 3.2.1. Factors related to the academic context

The most cited factor in the articles considered is that of the academic level of the students. Thus, Bolaños and Rodríguez (35), Haack (41), Quina-Galdino et al. (25) and Valero-Chilleron et al. (30) all observed an increase in one or more dimensions of burnout as students progressed through their course of study. By contrast, de Vasconcelos et al. (31) reported greater burnout in the second year, and Kong et al. (49), in the first years of study; similarly, Tomaschewski-Barlem et al. (29) recorded lower levels of personal accomplishment in the first years.

Aghajari et al. (45), Akansel et al. (46), and Kong et al. (49) all commented that an interest in nursing or the choice of this field of study as first option were significant factors in reducing susceptibility to burnout. In this respect, too, Tomaschewski-Barlem et al. (29) and De Vasconcelos et al. (31) found that burnout was more likely when the student was more inclined to abandon the study course. This conclusion was corroborated by Wang et al. (54), who reported that academic engagement was inversely related to the probability of burnout.

Other factors found to be related to burnout were the quality and flexibility of learning, and the problems that may arise from these considerations (34, 40, 45). Thus, the better the learning experience, the greater the flexibility and the fewer problems that arise, the lower the degree of burnout experienced by these students. Dissatisfaction with the academic course or with the practicum, the perception of a poor teaching environment or organization, or problems with the performance of theoretical or practical classes, all tend to aggravate burnout (25, 30, 34, 40).

The development of burnout may also be influenced by the students' interaction with their teachers and by the level of support received from them. According to Aghajari et al. (45), Haack (41) and Ríos-Rízquez et al. (26), the poorer the relationship and the less support and interaction perceived, the greater the risk of burnout.

Nursing students who are committed to a leisure activity or who are employed, in addition to their studies, tend to have higher levels of personal accomplishment (29). However, a greater burden of home study is associated with a greater propensity to burnout (25).

#### 3.2.2. Interpersonal factors

Among the sociodemographic variables considered, four were found to be particularly significant as risk/protection factors for burnout syndrome: age, sex, marital status and number of children.

Tomaschewski-Barlem et al. (29) reported that younger students presented higher levels of PA than their older counterparts. Similarly, Ríos-Risquez et al. (27) found that older students suffered more from EE. On the other hand, Gibbons et al. (40) pointed out that older and more experienced students were less inclined to use avoidance strategies and were more confident and able to seek and obtain the necessary support. All of these qualities help reduce burnout. According to Bolaños and Rodríguez (35), female students experience more burnout than men, but the latter are at greater risk of developing it. In this regard, Wang et al. (53), agreed that women tend to perceive less PA, while men are more prone to EE. Kong et al. (49) found that men presented higher levels of burnout. Finally, Aghajari et al. (45) reported that burnout was more prevalent among single students and those with children.

Physical activity is a protective factor against burnout (17) while the regular use of medication is a risk factor (31). Adversities suffered in childhood and the perception of economic stress are also risk factors for burnout (23, 44).

Many studies have examined the impact of psychological factors, and these factors are reported to have the strongest correlation with the three dimensions of burnout: EE, D and PA. Thus, Ríos-Rísquez et al. (27) reported that psychological dissatisfaction increased EE and D, while other studies have found that students affected by compassion fatigue and personal distress presented greater burnout; on the other hand, compassion satisfaction, life satisfaction and subjective sleep satisfaction are all protective factors (17, 36, 48, 50).

Personality type is another important factor. Students who have a resistant personality (with control, commitment and openness to challenge) and who are proactive are less affected by burnout (with lower levels of EE and D and greater PA) (37, 49). The importance of personality has also been highlighted (32, 37, 38), these authors used the NEO Five-Factor Inventory to study its correlation with each dimension of burnout. These authors detected a statistically significant relationship between neuroticism, EE and D, between agreeableness and D, between conscientiousness and all three dimensions of burnout, and between openness, D and PA.

Self-esteem, self-efficacy (general, in psychomotor skills, and in academic and professional contexts) and self-concept (of professionalism and as a care provider) have an indirectly proportional relationship with burnout. Thus, the higher the self-esteem, self-efficacy or self-concept, the lower the degree of burnout experienced (24, 28, 36, 47, 49, 53, 54).

The presence of anxiety and/or depression increases the risk of burnout and its development, especially in terms of EE and D (23, 36, 37, 42, 51). In this respect, too, Deary et al. (38) found that stress (affecting confidence and in financial, educational and clinical contexts) is another risk factor.

According to Haack (41), Naderi et al. (24) and Wang et al. (53), perceived emotional support, expressions of concern by other people, involvement with social networks, the need for cognition and the presence of leadership qualities are all protective factors against burnout. In addition, emotional intelligence (in dimensions such as conscience, control, empathy, motivation and social skills), together with emotional and cognitive empathy, are directly related to PA (17, 43). Finally, persons who are resilient are less susceptible to EE and D and have higher levels of PA (26, 27, 54).

Coping strategies can also influence the development of burnout (32, 38, 42). Thus, avoidant coping increases EE and reduces PA; problem-oriented coping increases PA and decreases D; and emotional coping increases EE, and according to Deary et al. (38) reduces PA. On the other hand, Katsifaraki et al. (42) conclude that emotional coping enhances PA.

Students who feel responsible for their behavior and responses (internal attribution) have a greater sense of PA, while those who attribute events to their surroundings, to the patient or to other people (external attribution) are more likely to suffer D and EE. According to another psychological theory, persons with a higher level of external locus present stronger degrees of burnout. Those who suffer from alexithymia, i.e. difficulty identifying and describing feelings, and who are prone to external attribution, are also at greater risk of developing burnout (41, 42, 44).

#### 3.2.3. Factors related to the environmental and/or social context

Wang et al. (53) reported that the strength of relationship with co-workers can influence susceptibility to burnout. Thus, feelings of belonging to a team and the existence of social interaction are protective factors against burnout (34, 42). The discrepancy between one's own values and those of the workplace, and the witnessing of incivility by nurses, tutors and/or other health professionals are also significantly related to EE and D, increasing them in each of these cases (33, 42). Akansel et al. (46), observed higher levels of burnout among students who performed practicums in institutions with hospitalized patients, versus those who cared for outpatients. According to Katsifaraki et al. (42) and Lopes and Nihei (17), workload is positively related to EE and D and inversely related to PA, while appropriate supervision at work and satisfactory perceived reward are both associated with reduced EE (42).

### 3.3. Meta-analytic estimates of the correlation between burnout and age, personality factors, resilience, and empathy

According to our meta-analysis, the variables significantly correlated with EE were neuroticism (*r* = 0.42. 95%CI 0.20, 0.61 *p* < 0.05) agreeableness (*r* = −0.13. 95%CI −0.24, −0.01 *p* < 0.05) and resilience (*r* = −0.38. CI95% −0.61, −0.10 *p* < 0.05).

Those correlated with D were agreeableness (*r* = −0.18. 95% CI −0.29, −0.06 *p* < 0.05), conscientiousness (*r* = −0.14. 95% CI −0.25,−0.02 *p* < 0.05) and resilience (*r* = −0.11. CI95% −0.22, −0.001 *p* < 0.05).

Those correlated with PA were conscientiousness (*r* = 0.18 95%CI 0.06, 0.29 *p* < 0.05), resilience (*r* = 0.40 95%CI 0.27, 0.60 *p* < 0.05) and empathy (*r* = 0.20 95%CI 0.11, 0.29 *p* < 0.05). Table 3 summarizes the results of the meta-analyses.

**Table 3:** Effect size (meta-analysis of correlation between burnout dimensions and other variables).

**Variable**	***n***	**Effect size r(95%CI)**	***I* ^2^**	***k***
**EE**				
Age	331	0,14 (−0.05, 0.32)	64.4%	2
Neuroticism	281	0.42 (0.20, 0.61)^*^	77.4%	2
Extraversion	281	−0.18 (−0.45, 0.11)	83.8%	2
Openness	281	−0.10 (−0.36, 0.18)	81.7%	2
Agreeableness	281	−0.13 (−0.24, −0.01)^*^	0%	2
Conscientiousness	281	−0.05 (−0.36, 0.27)	86.7%	2
Resilience	331	−0.38 (−0.61, −0.10)^*^	85.6%	2
Empathy	418	−0.009 (−0.10, 0.08)	0%	2
**D**				
Age	331	0,06 (−0.05, 0.16)	0%	2
Neuroticism	281	0.18 (−0.04, 0.38)	71.5%	2
Extraversion	281	−0.06 (−0.31, 0.19)	78.5%	2
Openness	281	−0.12 (−0.34, 0.11)	73%	2
Agreeableness	281	−0.18 (−0.29, −0.06)	0%	2
Conscientiousness	281	−0.14 (−0.25, −0.02)^*^	49%	2
Resilience	331	−0.11 (−0.22, −0.001)^*^	29.5%	2
Empathy	418	−0.19 (−0.43, 0.07)	84.6%	2
**PA**				
Age	331	−0,01 (−0.12, 0.10)	0%	2
Neuroticism	281	−0.10 (−0.22, 0.02)	0%	2
Extraversion	281	0.14 (−0.16, 0.41)	84.4%	2
Openness	281	−0.07 (−0.31, 0.18)	76.8%	2
Agreeableness	281	0.03(−0.09, 0.14)	0%	2
Conscientiousness	281	0.18 (0.06, 0.29)^*^	0%	2
Resilience	331	0.40 (0.27, 0.60)^*^	69.5%	2
Empathy	418	0.20 (0.11, 0.29)^*^	0%	2

CI, confidence interval; D, Depersonalisation; EE, Emotional exhaustion; n, Sample size; I2, Heterogeneity; k, Number of studies included in the meta-analysis. PA, Personal accomplishment; r, Correlation meta-analysis; ^*^p < 0.05.

## 4. Discussion

The aim of this study is to identify the factors that may affect the appearance and development of burnout in nursing students, in order to prevent or reduce its harmful effects. To date, this question has received relatively little research attention. The research papers we consider discuss a wide range of factors, the most important of which are sociodemographic, academic/occupational and psychological.

Within the academic context, several factors are related to burnout, including learning difficulties and the lack of facilitators (56). Moreover, EE is reported to be much higher in final-year students than in those at early stages of the university course (57). However, there is no clear consensus in this respect among the studies analyzed.

Other factors associated with academic burnout include insufficient interaction and relationship with teachers, a perceived lack of support, over-tasking, and the inadequate quality and organization of teaching, in areas such as timetables, the location of theoretical and practical classes, information on academic activities and library service management (55, 58). Among protective factors against burnout, the articles reviewed refer to academic commitment, interest in studying nursing and satisfaction with teachers (59).

In extreme cases, a loss of interest in studying (a factor closely related to the development of burnout) can lead students to abandon their course of studies (31, 60). Moreover, even after graduation, burnout can impel nurses to leave the profession (60).

The most influential sociodemographic variables in this context are age, sex, marital status and number of children. Cañadas-De la Fuente et al. (9) observed a significant relationship between D and marital status, finding that students who were single were more likely to suffer burnout. They also found a significant relationship between EE and the number of children. For nurses, having children is a protective factor, but in the case of students it is considered a risk factor. Male nurses (whether graduate or student) are at greater risk of developing burnout than their female counterparts. Although female students are more likely to experience greater burnout level than men (35), as well as female clinical nursing experience (9). Graduate nurses found that greater age was associated with lower EE, lower D and higher PA (61). However, Gradiski et al. (62) found no significant relationship between age and burnout in medical students.

Performing extracurricular activities, being in employment and doing physical exercise are all considered protective factors against burnout. Having better subjective health is also associated with greater resistance to burnout. By contrast, the use of medication, especially when used to control symptoms of anxiety and depression or to be able to sleep, is related to higher levels of burnout. The frequency with which the medication is taken is also significant; the greater the frequency, the higher the level of burnout (59).

In the psychological context, Fornés-Vives et al. (63) studied various groups of students in the field of health science and reported that neuroticism and coping strategies may be related to the development of burnout. In a similar study, Cañadas-De la Fuente et al. (64) reported that psychological factors such as neuroticism, agreeableness, openness and confidence are related to the development (or prevention) of burnout, both among students and among working professionals.

Personal adversities during childhood or collective ones (such as those provoked by an economic crisis) can provoke or aggravate burnout both in nursing students and in professionals. On a personal level, childhood traumas can cause symptoms of post-traumatic stress, producing fatigue and stress in the academic context (64). In the context of future employment, an economic crisis can seriously hamper graduates' entry into the labor market, an outlook that might reduce their present academic commitment (44, 65). In view of these considerations, it is readily understandable that adversities tend to provoke burnout and worsen academic performance (66).

Anxiety, both in general and specifically in pre-exam periods, together with low levels of perceived self-efficacy regarding the challenges that arise during university studies, are significant risk factors for burnout (55). Similarly, Hwang and Kim (59) observed a significant relationship between burnout, stress, anxiety and depression. Students who had not yet had completed their practicum were commonly subject to depression, while those who had, suffered both stress and depression, and hence were liable to experience burnout (67).

Compassion fatigue is a form of secondary stress that appears when the nurse's emotional capacity to cope with empathic commitment to the patient's suffering is overwhelmed. This problem is caused by prolonged contact with patients whose condition presents no appreciable improvement, a situation that can affect nursing staff at an emotional level, causing anguish, stress and burnout. On the other hand, satisfaction may be derived from compassion or from achievements in patient care, and this would be a protective factor against burnout (68). Nurses who work shifts may have sleep problems, be more exposed to stress and have less job satisfaction, in comparison with those who work a fixed schedule. The latter are more likely to have satisfactory sleep patterns and to be happier with their work. This would generate compassion satisfaction, acting as a protective factor against burnout and hence improving patient care (53, 69, 70).

Negative emotional experiences in nursing students may cause nervousness, anxiety and even fear, producing cognitive, physiological and behavioral changes. Moreover, these impacts are often aggravated by stress, leading those affected to employ negative coping strategies such as avoidance. It is generally agreed that students who only achieve emotional coping by means of avoidance have greater difficulty in adapting to problematic situations, which may impact on their academic and professional performance. Moreover, the greater the number of stressful situations experienced, the higher the degree of coping needed, with potentially serious consequences (71, 72).

In psychology, attribution is defined as the process by which the causes of events or behaviors are inferred. Intervention therapies such as mindfulness can limit the extent of stress and academic burnout and help those affected become more aware in their daily lives (73).

When students experience incivil behavior in an academic or clinical setting, they may believe it to be an unalterable aspect of nursing culture that they must endure when they become professionals (74). Such behavior by their mentors may be due to a stressful situation being experienced or to a lack of teaching preparation/ability (75). Unfortunately, this outcome is exacerbated when students, too, are subjected to stress (76) or if they express discrepancies with their mentors, perhaps due to generational change (77).

In the future, interventions should be designed and carried out to increase the empathy and resilience of students, especially those most susceptible to stress. Success in this approach would enhance compassion satisfaction and decrease burnout (68). In this respect, the strategies likely to have the greatest impact would be those aimed at correcting avoidance coping and at enhancing adaptive coping. It would also be advisable to reinforce family support, in order to reduce compassion fatigue and to facilitate compassion satisfaction in students during their clinical practice (54, 78).

Nursing students who developed burnout throughout their career will be more likely to experience burnout during their clinical work. For this reason, it is essential that students acquire the necessary skills to prevent this mental health problem (77).

If we focus our results on clinical nursing, poor management can worsen the work environment for nurses and hence the quality of employment. For this reason, nursing managers should promote the detection burnout syndrome in nurses and promote interventions for the prevention of this syndrome (79, 80).

This study has some limitations. Some results should be considered with caution, because the number of included studies is not high, the studies have been done in different countries with different nursing education and the generational differences between students should be taken into account. Thus, more research should be done focusing on this topic in the future.

## 5. Conclusion

The question of preventing burnout in nursing students should be addressed from the outset of their studies. Coping methods and resources should be fostered as an integral part of the educational framework, in order to promote the acquisition of resilience, agreeableness, empathy and conscientiousness. Professors should teach nursing students to prevent and recognize the most frequent symptoms of burnout syndrome, particularly to those younger, with children and neuroticism personalities. So that, these students in risk could recognize when they are burning and can request for psychological help.

## Data availability statement

No restrictions applied. Requests to access the datasets should be directed to mariajose.membrive@gmail.com.

## Author contributions

Conceptualization: AV-S and NS-M. Methodology: LP-H and LR-B. Software: JG-U. Validation: JG-U, NS-M, and GC-D. Formal analysis: JG-U and MM-J. Investigation: MM-J and LR-B. Resources, data curation, and writing—original draft preparation: AV-S and MM-J. Writing—review and editing: LP-H, LR-B, and NS-M. Visualization: LR-B and AV-S. Supervision: JG-U and GC-D. Project administration: LP-H and GC-D. All authors have read and agreed to the published version of the manuscript.
